# Immunosuppressive therapy in virus-negative inflammatory cardiomyopathy: 20-year follow-up of the TIMIC trial^[Author-notes ehac348-FM1]^

**DOI:** 10.1093/eurheartj/ehac348

**Published:** 2022-07-14

**Authors:** Cristina Chimenti, Matteo Antonio Russo, Andrea Frustaci

**Affiliations:** Department of Clinical, Internal, Anesthesiology and Cardiovascular Sciences, Sapienza University of Rome, Rome, Italy; Molecular and Cellular Cardiology Lab, IRCCS ‘L. Spallanzani’, Rome, Italy; MEBIC Consortium, San Raffaele 21 University, Rome, Italy; Department of Clinical, Internal, Anesthesiology and Cardiovascular Sciences, Sapienza University of Rome, Rome, Italy; Molecular and Cellular Cardiology Lab, IRCCS ‘L. Spallanzani’, Rome, Italy

**Keywords:** Inflammatory cardiomyopathy, Myocarditis, Immunosuppressive therapy, Follow-up, TIMIC

## Abstract

**Aims:**

Long-term results of the Tailored IMmunosuppression in virus-negative Inflammatory Cardiomyopathy (TIMIC) trial protocol have been evaluated.

**Methods and results:**

Eighty-five patients with endomyocardial biopsy-proven virus-negative chronic inflammatory cardiomyopathy were enrolled in the randomized, double-blind, placebo-controlled TIMIC trial and received prednisone and azathioprine (*n* = 43) vs. placebo (*n* = 42) for 6 months. Immunosuppressive treatment promoted an improvement in cardiac function in 88% of the cases compared with none of the patients in the placebo group, which were switched to a 6-month immunosuppressive therapy at the end of the 6-month study period. Long-term (up to 20 years) clinical outcomes of the whole cohort of 85 patients originally enrolled in the TIMIC trial (Group A) were compared with those of a 1:2 propensity score-matched control cohort of patients untreated with the TIMIC protocol (Group B) and followed for a comparable period of time. The primary outcome was a composite of cardiovascular death and heart transplantation. At long-term follow-up, the risk of cardiovascular death [hazard ratio (HR) 6.77; 95% confidence interval (CI) 2.36–19.45] and heart transplantation (HR 7.92; 95% CI 1.80–34.88) was significantly higher in Group B patients. Group A showed a persistent improvement in the left ventricular ejection fraction compared with Group B (HR 7.24; 95% CI 3.05–17.18). A higher number of Group B patients underwent implantable cardioverter defibrillator implantation. The incidence of recurrent myocarditis was similar between groups, and patients with evidence of a recurrent cardiac inflammatory process promptly responded to a TIMIC protocol application.

**Conclusion:**

Virus-negative inflammatory cardiomyopathy benefits from immunosuppressive therapy even after long-term follow-up. Recurrence appears to respond to a new TIMIC protocol application.

## Structured Graphical Abstract

**Structured Graphical Abstract:**
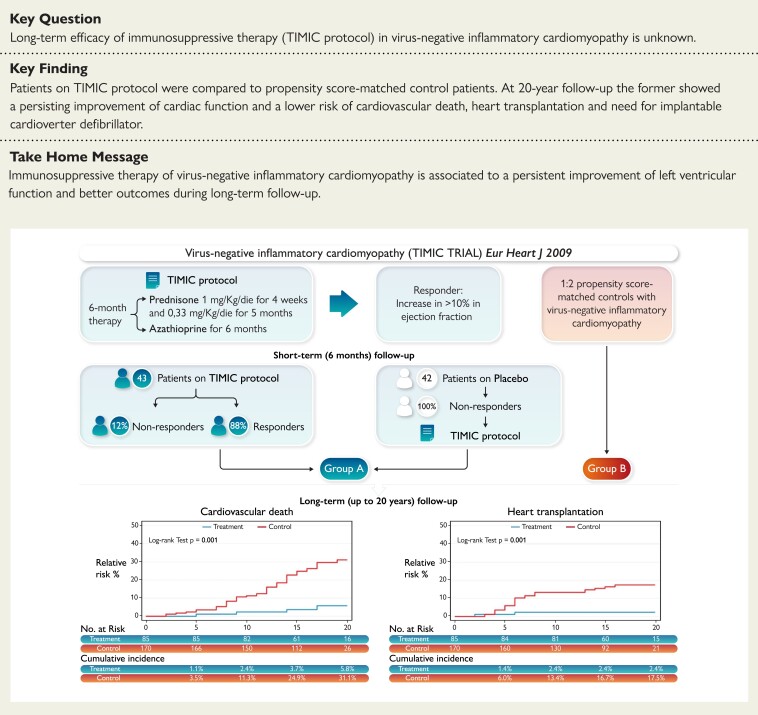
Box plots of the distribution of left ventricular ejection fraction and left ventricular end-diastolic volume at baseline, short-term (6 months), and long-term follow-up in patients on TIMIC protocol (blue) and control patients (green) are presented in the left upper panel. The composite endpoint of cardiovascular death and heart transplantation (primary outcome) during follow-up in patients on TIMIC protocol (blue line) and control patients (red line) is shown in the left lower panel. The incidence of cardiovascular death (right upper panel) and heart transplantation (right lower panel) during follow-up in patients on TIMIC protocol (blue line) and controls (red line) is also presented.

**See the editorial comment for this article ‘Advanced diagnostics in inflammatory cardiomyopathy for personalized therapeutic decision-making’, by Heinz-Peter Schultheiss and Felicitas Escher, https://doi.org/10.1093/eurheartj/ehac412.**

## Introduction

Myocarditis is an inflammatory disease of the myocardium that can manifest as a wide range of clinical features, including acute or chronic heart failure (HF), brady- and tachyarrhythmias, or, occasionally, sudden cardiac death.^1^

Acute HF due to active myocarditis can be a dramatic event that may require a prompt circulatory support with inotropes or mechanical devices in fulminant cases.^2^ Acute phase survivors may either have a rapid systolic functional recovery or progress to an end-stage disease, sometimes requiring cardiac transplantation. In the latter scenario, there was evidence of a virus-negative immune-mediated pathway that contributed to the formulation of the Tailored IMmunosuppression in virus-negative Inflammatory Cardiomyopathy (TIMIC) protocol, which was an immunosuppressive therapeutic strategy based on the combination of prednisone and azathioprine for 6 months.^3^ This approach has been demonstrated to successfully improve cardiac dimensions and function in 88% of treated patients enrolled in the randomized TIMIC trial. Of note, this 6-month immunosuppressive regimen led to a significant increase in the left ventricular ejection fraction (LVEF) even in patients with long-standing severe left ventricular dilation and dysfunction.^3^

Further studies from several groups have confirmed the efficacy of immunosuppressive therapy in patients with biopsy-proven virus-negative inflammatory cardiomyopathy.^4–11^

Nonetheless, little is known about the long-term implications of 6-month immunosuppression in cardiac structure/function, as well as the incidence of relapsing myocardial inflammation over time.^12^ In this perspective, it can be postulated that a new TIMIC protocol application may be potentially beneficial in the case of recurrent myocarditis.

The aim of the present report is to describe the long-term outcomes of patients originally enrolled in the TIMIC trial, the incidence of relapsing myocarditis, and its response to a new TIMIC protocol cycle.

## Methods

The current study population consists of 85 patients (51 men and 34 women, mean age of 42.7 ± 15.4 years) originally enrolled in the randomized, double-blind, placebo-controlled TIMIC trial.^3^

In the original study,^3^ patients were enrolled between January 2001 and January 2007 and randomly assigned to one of the two treatment groups: oral administration of immunosuppressive therapy (43 patients, Group 1) including prednisone (1 mg/kg daily for 4 weeks followed by 0.33 mg/kg daily for 5 months) and azathioprine (2 mg/kg daily for 6 months) or placebo (42 patients, Group 2). All patients had complained about symptoms of HF of unknown cause for at least 3 months, despite optimal conventional therapy with angiotensin-converting enzyme inhibitors, beta-adrenergic blocking drugs, and diuretics. There were no significant differences in baseline characteristics between groups. All patients had undergone baseline biopsy; the diagnosis of myocarditis was achieved according to the Dallas criteria and confirmed by immunohistochemistry. Polymerase chain reaction (PCR) and reverse transcriptase–PCR analysis were performed on frozen sections to exclude the presence of cardiotropic viruses. The study protocol included cardiac catheterization, angiography, and biventricular endomyocardial biopsy at baseline and at 6 months. At 6 months from enrollment, all patients allocated to the placebo group showed persistent/worsening cardiac dysfunction and were prescribed the 6-month TIMIC protocol as a result of the superiority of this immunosuppressive strategy vs. placebo documented in the trial.

In the current study, the entire group of 85 TIMIC trial patients who had undergone immunosuppressive therapy (Group A, experimental group) was compared with a 1:2 propensity score-matched group of patients who had not received immunosuppressive therapy (Group B, matched control group).^3^ Control patients were selected via propensity score matching (PSM) among all patients admitted to our institution between June 2000 and December 2005 who had an endomyocardial-biopsy-proven diagnosis of virus-negative chronic inflammatory cardiomyopathy and were never prescribed immunosuppressive therapy.^13^ No clinical or histological differences regarding the burden of fibrosis and number of CD3-positive cells were detected between Group A and Group B. Group B did not receive any immunosuppressive therapy due to patient refusal to participate in the TIMIC study or clinical onset before TIMIC trial results. All patients were treated with optimal conventional HF therapy. Group A and Group B patients had a long-term follow-up of up to 20 years.

The study complies with the Declaration of Helsinki, the locally appointed ethics committee approved the research protocol, and informed consent was obtained from all subjects.

### Clinical studies and follow-up

Long-term effectiveness was assessed in all patients on a yearly basis; routine follow-up visits included clinical evaluation (physical examination and routine laboratory tests) and non-invasive cardiac studies (ECG and 2D ecocardiography). The New York Heart Association (NYHA) class was used to assess functional capacity, which was determined by a questionnaire.

Echocardiographic studies were performed with Agilent Sonos 5500 (Hewlett-Packard, Palo Alto, CA, USA) (from 2001 to 2013) and with Model P7 (General Electrical Medical, Chicago, IL, USA) (from 2014 to 2021). Patients were imaged, and data were analysed offline by senior echocardiographers. Echocardiographic parameters were determined according to the established criteria.^14^ In particular, the ejection fraction was calculated in the apical four- and two-chamber views from three separate cardiac cycles using the modified Simpson’s method. Cardiac magnetic resonance imaging (MRI) at baseline and short-term follow-up was not performed systematically and, therefore, not used for comparison in the present study. However, patients with recurrence of myocarditis during long-term follow-up were prescribed cardiac MRI, as previously described.^15^

An additional endomyocardial biopsy during the long-term follow-up was considered in case of severe worsening of cardiac function despite full conventional HF treatment. Five to seven samples were drawn from the septal apical region of the left ventricle as previously described^16^ and were processed for histology, immunohistochemistry, transmission electron microscopy, and molecular biology.

In particular, the presence of 14 infiltrating leucocytes/mm^2^ and/or the presence of more than 2.0 CD3-positive lymphocytes per high power field, often adherent to the contour of cardiomyocytes and focally associated with cell necrosis, were considered diagnostic for myocarditis.

In recurrent myocarditis, immunohistochemistry for TLR4 was used to detect the presence of autoimmune activation of cardiomyocytes, according to the evidence that myocardial TLR4 expression is a useful tool to discriminate responders vs. non-responders to immunosuppression.^17^ Two frozen myocardial specimens from each patient were used for real-time PCR analysis to detect the presence of cardiotropic viruses, including adenovirus, Epstein–Barr virus, human herpesvirus 6, parvovirus B19, herpes simplex virus 1–2, cytomegalovirus, enterovirus, influenza A and B viruses, and hepatitis C virus.^3^

### Clinical outcome

The primary outcome was a composite of cardiovascular death and heart transplantation.

Secondary outcomes included each individual outcome of the primary composite outcome plus left ventricular systolic function changes [i.e. LVEF and left ventricular end-diastolic volume (LVEDV) at echocardiography] over time. Specifically, we classified patients as improved if they showed a >10% increase in the absolute LVEF, as in the TIMIC trial.

In addition, we evaluated the rate of myocarditis relapse and the number of patients who underwent implantable cardioverter defibrillator (ICD) implantation.

### Statistical analysis

Propensity score matching was performed to reduce the risk of selection bias. Patients were divided into two cohorts: patients treated with the TIMIC protocol (experimental group) and patients not treated with the TIMIC protocol (matched control group). Due to differences in key baseline characteristics, and echocardiographic and electrocardiographic parameters, we used PSM for the two cohorts and assembled a cohort for each comparison; all the measured covariates were well-balanced across comparator groups. The propensity score is defined as the subject’s probability of receiving a specific treatment or exposure (in this case, the TIMIC protocol) given a set of measured baseline covariates.^18^ A logistic regression model was used to obtain propensity scores with the TIMIC protocol defined as the dependent variable, and age, gender, clinical characteristics, and echocardiographic parameters entered as covariates. Matching was performed using the nearest neighbour matching protocol (matching ratio of 1 to 2 without replacement) and a caliper width of 0.01. Assessment of balance in baseline characteristics was performed by estimating standardized differences between groups; standardized difference indicates the degree of systematic differences in covariates between groups. Operationally, a standardized difference >10% represents a meaningful imbalance in a given variable between groups.

Normal distribution of variables was assessed with the Kolmogorov–Smirnov test. For continuous variables, descriptive statistics were provided (number of available observations, mean, and standard deviation), while the median (interquartile range) was used for non-normal data. Categorical data were presented as numbers (percentage). Student’s *t*-test, the χ^2^ test, and the Fisher exact test were used for comparison. For all tests, a *P*-value of <0.05 was considered statistically significant.

The Kaplan–Meier method was used to estimate cumulative event rates in the two groups. Differences in each group were compared using log-rank tests. The Cox regression hazard model was performed to obtain the hazard ratio (HR) for the primary and secondary endpoints.

All statistical analyses were performed using STATA statistical analysis software (version 16).

## Results

### Clinical studies and follow-up

Clinical and echocardiographic data of patients enrolled in the two groups at baseline and long-term follow-up are summarized in *Tables 1* and *2*.

**Table 1:** Baseline patient characteristics by TIMIC protocol use before and after propensity score matching

	Non-matched groups			Matched groups		
	Treatment (*n* = 85)	Control (*n* = 517)	*P*-value	Treatment (*n* = 85)	Control (*n* = 170)	*P*-value
**Clinical characteristics**						
Age, years	44.6 ± 12.7	48.9 ± 11.4	**<0.001**	44.6 ± 12.7	43.8 ± 12.1	0.65
Male sex, *n* (%)	51 (60)	328 (63.4)	0.55	51 (60.0)	99 (58.2)	0.89
Hypertension, *n* (%)	2 (2.4)	58 (11.2)	**0**.**02**	2 (2.4)	11 (6.5)	0.23
Autoimmune disorder, *n* (%)	5 (5.9)	79 (15.3)	**0**.**03**	5 (5.9)	14 (8.2)	0.62
**NYHA class, *n* (%)**						
I	0	24 (4.6)	**<0.001**	0	1 (0.6)	1
II	48 (56.5)	186 (36.0)	**<0.001**	48 (56.5)	84 (49.4)	0.35
III	27 (31.8)	203 (39.3)	0.23	27 (31.8)	60 (35.3)	0.67
IV	10 (11.7)	104 (20.1)	0.07	10 (11.7)	25 (14.7)	0.57
**Electrocardiographic**						
AF, *n* (%)	8 (9.4)	96 (18.6)	**0**.**04**	8 (9.4)	23 (13.5)	0.42
LBBB, *n* (%)	15 (17.6)	156 (30.2)	**0**.**02**	15 (17.6)	32 (18.8)	0.87
**Echocardiographic**						
LVEF, %	25.9 ± 4.6	28.1 ± 6.1	**<0.001**	25.9 ± 4.6	26.5 ± 5.1	0.49
LVEDD, mm	67.4 ± 4.4	69.2 ± 5.3	**<0.001**	67.4 ± 4.4	68.0 ± 3.8	0.29
LVEDV, mL	243 ± 51	256 ± 53	**<0.001**	243 ± 51	251 ± 40	0.16

Bold values indicates statistically significant values (*p* < 0.05).

AF, atrial fibrillation; LBBB, left bundle branch block; LVEDD, left ventricular end-diastolic diameter; LVEDV, left ventricular end-diastolic volume; LVEF, left ventricular ejection fraction; NYHA, New York Heart Association.

**Table 2:** Clinical, electrocardiographic, and echocardiographic characteristics of TIMIC (treatment) and control patients at long-term follow-up.

	Treatment (*n* = 85)	Control (*n* = 170)	*P*-value
**Clinical characteristics**			
Age, years	61.1 ± 12.5	60.4 ± 11.8	0.69
Hypertension, *n* (%)	6 (7.1)	28 (16.5)	0.05
Autoimmune disorder, *n* (%)	5 (5.9)	18 (10.6)	0.25
**NYHA class, *n* (%)**			
I	59 (69.4)	67 (39.4)	**<0**.**001**
II	23 (27.1)	44 (25.9)	0.88
III	2 (2.4)	34 (20.0)	**<0**.**001**
IV	1 (1.1)	25 (14.7)	**<0**.**001**
**Electrocardiographic**			
AF, *n* (%)	0	38 (22.4)	**<0**.**001**
LBBB, *n* (%)	4 (4.7)	54 (31.8)	**<0**.**001**
**Echocardiographic**			
LVEF, %	50.2 ± 9.8	26.9 ± 7.0	**<0**.**001**
LVEDD, mm	56.7 ± 4.6	67.4 ± 6.8	**<0**.**001**
LVEDV, mL	145.4 ± 47.6	231.4 ± 32.4	**<0**.**001**

Bold values indicates statistically significant values (*p* < 0.05).

AF, atrial fibrillation; LBBB, left bundle branch block; LVEDD, left ventricular end-diastolic diameter; LVEDV, left ventricular end-diastolic volume; LVEF, left ventricular ejection fraction; NYHA, New York Heart Association.

**Figure 1:**
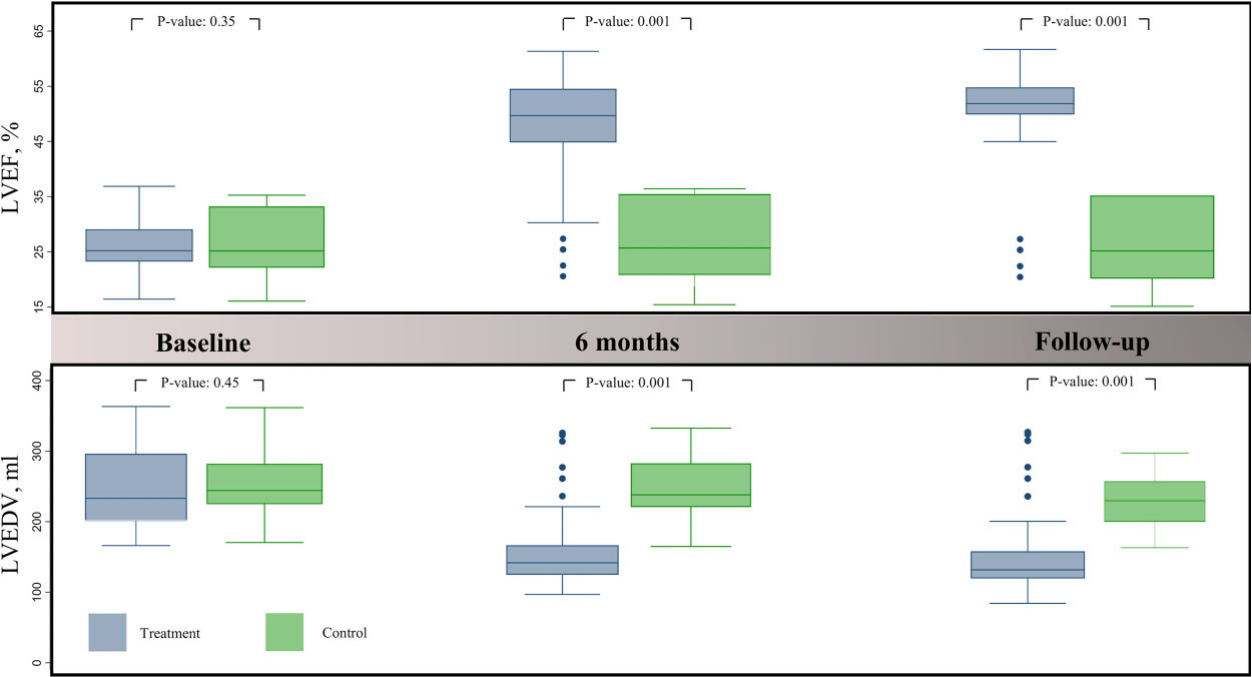
Box plots of the distribution of left ventricular ejection fraction and left ventricular end-diastolic volume at baseline, short-term (6 months), and long-term follow-up in Group A (left) and Group B (right) patients. LVEF, left ventricular ejection fraction; LVEDV, left ventricular end-diastolic volume.

At baseline, the mean LVEF for the two groups of patients (Group A: treatment patients; Group B: control patients) was comparable (*P* = 0.49), and most patients were in NYHA Class III or IV. Six months after immunosuppressive treatment, Group A patients showed a significant improvement in LVEF and a reduction in LVEDV compared with baseline; overall, 96.5% of patients were in NYHA Class I and II. This effect persisted over a long-term follow-up period (HR 7.24; 95% CI 3.05–17.18) (*Figure 1*) and was also documented in patients with severe left ventricular dilation and dysfunction at baseline. Conversely, no significant changes in LVEF occurred in Group B patients during a short- and long-term follow-up period. Overall, Group A had a significantly higher LVEF than Group B at long-term follow-up.

Remarkably, Group A patients, who were originally on placebo in the TIMIC trial and were subsequently switched to immunosuppression, showed an improvement similar to that observed in the group originally randomized to immunosuppressive therapy either at short- or long-term follow-up.

The most common electrocardiographic findings were repolarization abnormalities that normalized in case of recovery. Notably, 13 Group A patients had left bundle branch block at onset, which regressed to normal or nearly normal intraventricular conduction in 9 cases after short-term immunosuppression; this normalization persisted over time. Eight Group A patients had atrial fibrillation that was electrically or pharmacologically cardioverted; this arrhythmia did not relapse after recovery from myocarditis.

Major adverse events during long-term follow-up are reported in *Table 3*.

**Table 3:** Major adverse events during follow-up in the two groups.

Outcome	Treatment (*n* = 85)			Control (*n* = 170)			*P*-value
	Patients with events	Events	Events/100 p-y	Patients with events	Events	Events/100 p-y	
**Composite endpoint**	6 (7.1)	6	0.4	70 (41.2)	75	3.1	**<0.001**
CV death	4 (4.7)	4	0.3	48 (28.2)	48	1.8	**<0.001**
Heart transplantation	2 (2.4)	2	0.1	27 (15.9)	27	1.2	**0.003**
ICD implantation	9 (10.6)	9	0.1	72 (42.4)	72	3.5	**<0.001**
Recurrence	5 (5.9)	5	0.4	14 (8.2)	14	0.6	0.62
Follow-up, years	**16.6** ± **2.9**			**15.8** ± **3.8**			**0**.**11**

Bold values indicates statistically significant values (*p* < 0.05).

CV, cardiovascular death; ICD, implantable cardioverter defibrillator; p-y, patient-years.

A higher number of patients in Group B underwent ICD implantation [Group A: 9 (10.6%) vs. Group B: 72 (42.7%); *P* < 0.001]. The incidence of relapsing myocarditis was similar between groups [Group A: 5 (5.9%) vs. Group B: 14 (8.2%); *P* = 0.62].

**Figure 2:**
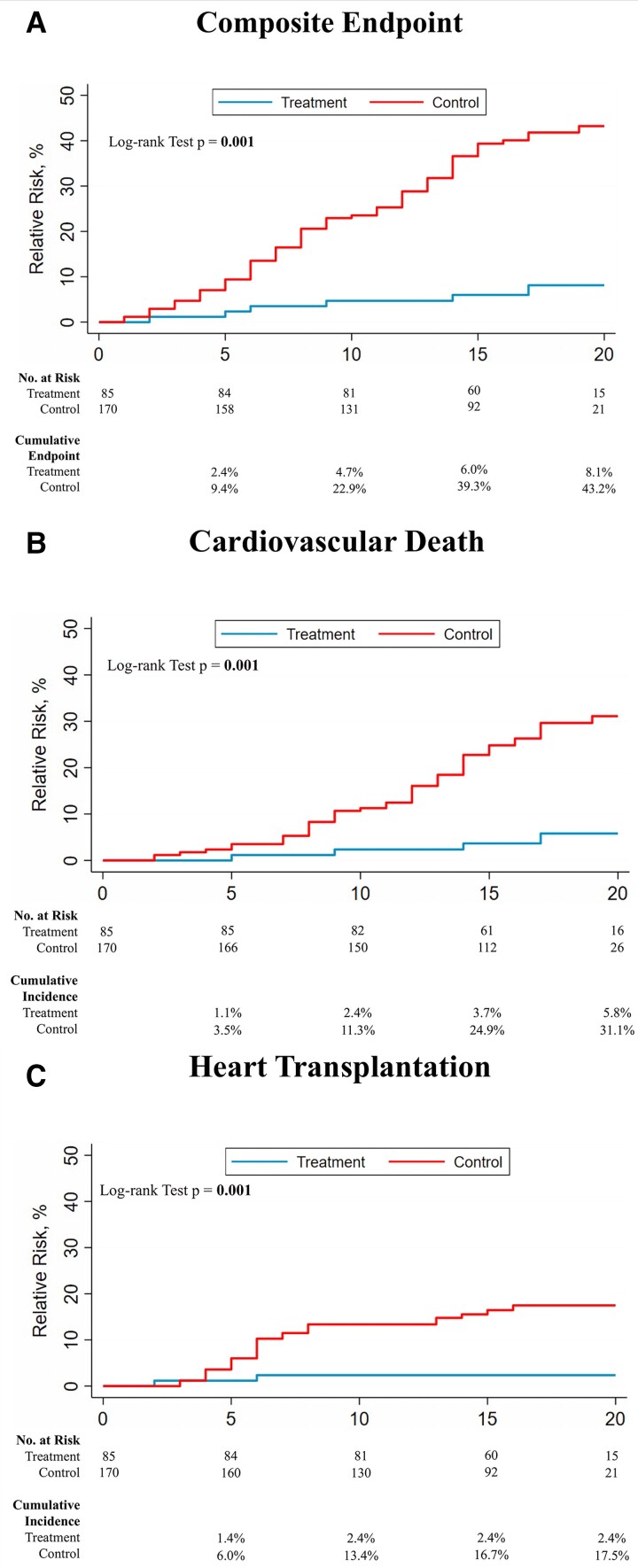
Composite endpoint of cardiovascular death and heart transplantation (primary outcome) (*A*), the incidence of cardiovascular death (*B*), and heart transplantation (*C*) during follow-up in Group A (left line) and Group B (right line) patients.

The cumulative incidence of recurrence, composite endpoint, heart transplantation, and death during follow-up is shown in *Figure 2*.

**Figure 3:**
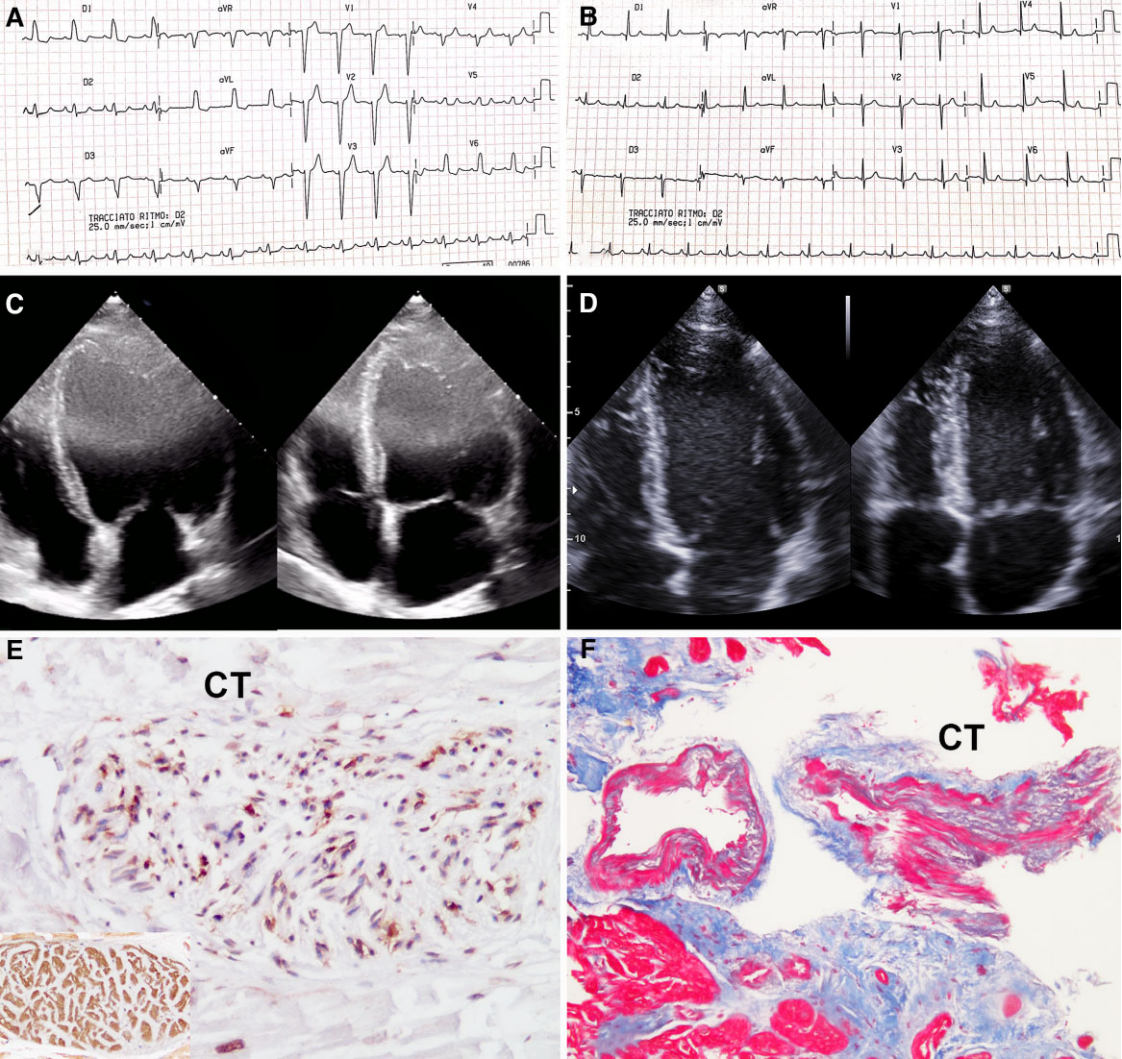
Recurring virus-negative myocarditis responding to immunosuppressive therapy (Patient 5). Electrocardiogram showing left bundle branch block (*A*) resolving after therapy (*B*). (*C* and *D*) Echocardiographic apical view showing recovery of left ventricular dysfunction [ejection fraction from 28% (*C*) up to 51%]. (*E* and *F*) Unusual inclusion of segment of conduction tissue showing inflammation (CD45 RO, immunoperoxidase 20×, square: conduction tissue-specific HCN4 immunostaining) resolving after immunosuppressive therapy (Masson trichrome, 10× magnification).

**Figure 4:**
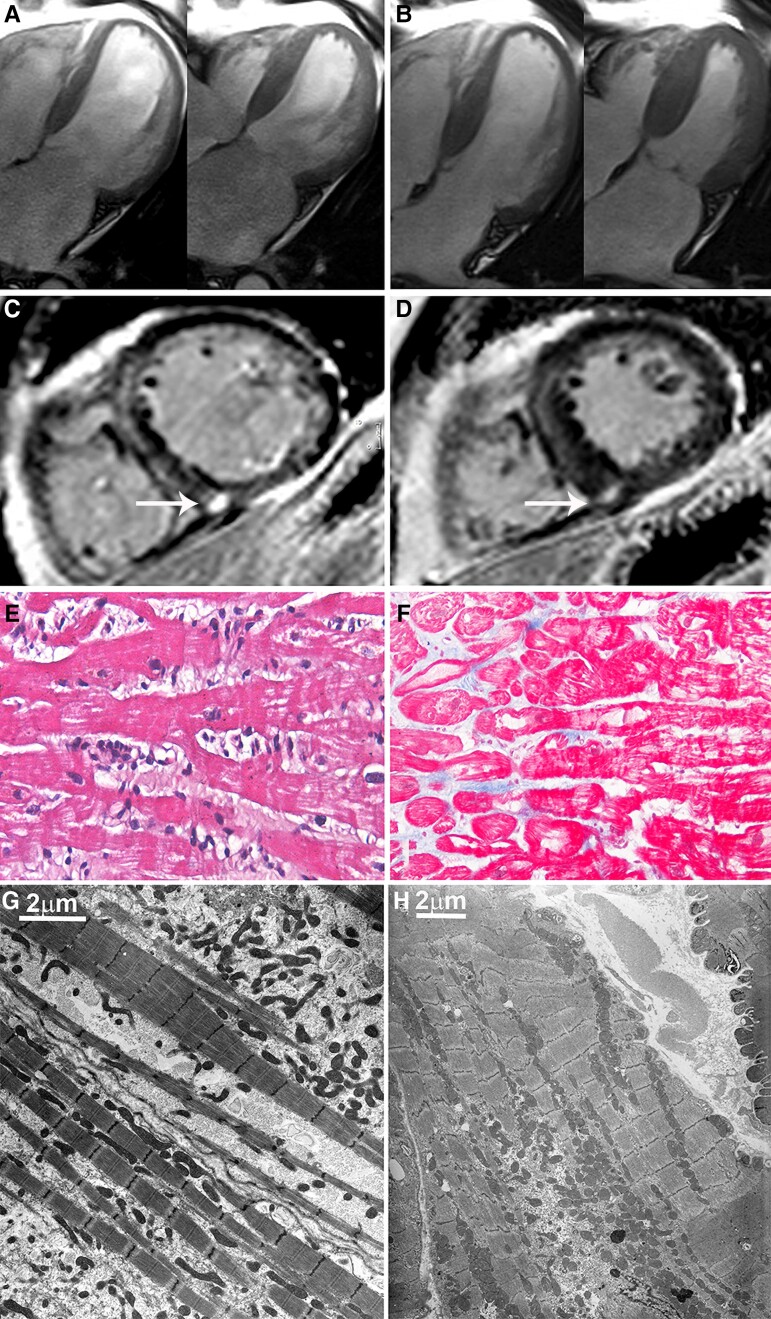
Recurrence of myocarditis responding to TIMIC protocol treatment (Patient 1). Cardiac magnetic resonance four-chamber apical view showing severe reduction of cardiac function (*A*—left ventricular ejection fraction 30%) that improves after therapy (*B*—left ventricular ejection fraction 55%). A diffuse and severe myocardial damage was revealed on contrast-enhanced images as an extensive and nuanced late enhancement of the entire ventricular wall (*C*), with predominant involvement of the mid-wall layer of the interventricular septum and the subendocardial layer of the anterolateral wall, and focal area of greater enhancement at the inferior interventricular junction reflecting focal replacement fibrosis (arrow). After 6 months of treatment, late gadolinium enhancement was less extensive and more slight (*D*) as an expression of damage regression. Histology showed an active lymphocytic myocarditis (*E*, haematoxylin–eosin 20× magnification) that regressed to a healed phase (*F*, Masson trichrome, 20× magnification) after treatment. (*G*) Transmission electron microscopy from the same patient: before treatment, areas of myofibrillolysis are evident, occupied by cytosolic components. After immunosuppressive treatment (*E*), the empty cytosolic areas disappeared, and the overall ultrastructure appears to be similar to a normal myocardium (scale bar = 2 μm).

Five Group A patients (6%) experienced a worsening of cardiac function in the long term (9.8 ± 2.3 years). The characteristics of these patients are described in *Table 4*. Four of them (4F, 53 ± 8.6 years) had a history of autoimmune diseases (i.e. Hashimoto thyroiditis in three cases and autoimmune piastrinopenia in one case); the remaining patient (M, 70 years) had a recurrence after 13 years following a flu-like syndrome. Cardiac MRI was suggestive of myocarditis, according to the Lake Louis criteria.^15^ In particular, tissue oedema was present in 60% of patients, while hyperaemia and late gadolinium enhancement, suggestive of fibrosis, were present in all patients and were mainly located in the mid-lateral basal segment of the left ventricle with either a mid-wall or sub-epicardial pattern of distribution. All five patients underwent a new endomyocardial biopsy after providing informed consent. No periprocedural complications were observed. Histology and immunohistochemistry showed a reactivation of the inflammatory process (*Figures 3* and *4*) and transmission electron microscopy revealed areas of myofibrillolysis (*Figure 4*) occupied by cytosolic components. In one patient (n. 5 in *Table 4*) with a left bundle branch block, inflammation of the conduction tissue was demonstrated at endomyocardial biopsy (*Figure 3*). None of them were positive for cardiotropic viruses. Patients were prescribed immunosuppressive therapy with the same 6-month TIMIC protocol.

**Table 4:** Clinical, echocardiographic, and immunohistological characteristics of the five TIMIC patients with recurrence of myocarditis at follow-up

	Patient 1	Patient 2	Patient 3	Patient 4	Patient 5
**Recurrence**					
**Demographics**					
Age, years	70	48	52	69	44
Sex	M	F	F	F	F
Time to relapse, years	13	8	11	10	7
**Clinical**					
Hypertension	Yes	No	No	Yes	No
LBBB	No	No	No	No	Yes
Autoimmune disorder	No	Yes	Yes	Yes	Yes
NYHA class	III	III	II	III	III
**Echocardiographic**					
LVEF, %	30	25	20	34	26
LVEDD, mm	60	70	64	67	68
LVEDV, mL	245	245	240	210	225
**CMR findings**					
LVEF, %	27	23	21	35	28
LVEDV, mL	239	211	234	198	213
Oedema	No	Yes	Yes	Yes	No
Early gadolinium enhancement	Yes	Yes	Yes	Yes	Yes
Late gadolinium enhancement	Yes	Yes	Yes	Yes	Yes
**6-month follow-up**					
**Echocardiographic**					
LVEF, %	55	50	50	56	51
LVEDD, mm	53	49	51	48	47
LVEDV, mL	126	136	140	129	132
**CMR findings**					
LVEF, %	53	51	53	54	54
LVEDV, mL	161	136	140	129	132
Oedema	No	No	No	No	No
Early gadolinium enhancement	No	No	No	No	No
Late gadolinium enhancement	Yes	Yes	Yes	Yes	Yes

The variables are reported at the time of recurrence of myocarditis and at the end of the second 6-month cycle of immunosuppression.

CD, cluster of differentiation; CMR, cardiac magnetic resonance; LBBB, left bundle branch block; LVEDD, left ventricular end-diastolic diameter; LVEDV, left ventricular end-diastolic volume; LVEF, left ventricular ejection fraction; NYHA, New York Heart Association, TLR4, Toll-like receptor 4.

Clinical assessment, resting ECG, and 2D echocardiography were performed at baseline, weekly during the first month, and every 4 weeks for the remaining 5 months. Control cardiac MRI, cardiac catheterization, angiography, and left ventricular endomyocardial biopsy were performed at 6-month follow-up (*Table 4*). Cardiac MRI showed a significant improvement in cardiac function with the disappearance of tissue oedema and hyperaemia and persistence in the areas of fibrosis. Control biopsy showed a resolution of the inflammatory process. Transmission electron microscopy showed an increase in the myofibrillar content compared with the first biopsy and the disappearance of areas of myofibrillolysis (*Figure 4*). Of note, the efficacy of immunosuppression on inflammation and cardiac function did not differ between first-time and relapsing myocarditis.

The 14 Group B patients who experienced a recurrent myocarditis had, in 64% of cases, an associated immuno-mediated disease and were treated with the TIMIC protocol as well.

## Discussion

### Immunosuppressive treatment in inflammatory cardiomyopathy

The position statement of the European Society of Cardiology recommends the individualized use of immunosuppression in infection-negative lymphocytic myocarditis refractory to standard therapy, as well as in patients with proven autoimmune forms of myocarditis (e.g. giant cell myocarditis, cardiac sarcoidosis, some forms of eosinophilic and toxic myocarditis, and myocarditis associated with known extra-cardiac autoimmune diseases).^1^ Several studies and meta-analyses have confirmed the usefulness of immunosuppressive treatment in selected patient populations with myocarditis.^3–11^ In particular, a meta-analysis of randomized controlled trials comparing 342 patients on immunosuppression with 267 patients on conventional therapy demonstrated a significant improvement in LVEF at both short-term (≤3 months) and intermediate-term follow-up (up to 2 years).^10^ A more recent analysis of prospective and retrospective studies showed lower mortality and improved cardiac function with immunosuppression, especially when patients were diagnosed with virus-negative biopsy-proven immune-mediated myocarditis.^5^

Immunosuppression should be started only after ruling out any active infection on endomyocardial biopsy by PCR analysis. Indeed, a retrospective study performed on patients on immunosuppressive treatment prescribed without preliminary viral genome search showed that those with a myocardial viral infection were unresponsive to the treatment.^19^ The randomized, double-blind, placebo-controlled TIMIC trial was designed as a result of the findings of this retrospective study; its findings demonstrated a positive impact of immunosuppression on left ventricular function recovery in a high proportion (88%) of patients. Remarkably, a striking improvement occurred even in patients with extreme left ventricular dilatation and dysfunction; these findings suggested a long-lasting history of the disease and were associated with a concomitant disappearance of inflammatory infiltrates with the progression of the disease from an active towards a healed form at histological examination. Moreover, arrhythmia control, as well as conduction system (i.e. left bundle branch block) functional recovery, resulting in a restored biventricular synchrony,^20^ were also documented, while no deaths or cardiac transplantation occurred during the 6 months of the trial (short-term follow-up).

### Long-term efficacy of immunosuppressive treatment

In the present study, we reported the long-term data of patients originally recruited in the TIMIC trial. This is the first study on immunosuppression in inflammatory cardiomyopathy, describing the long-term efficacy of this treatment on cardiac dimension and function and on HF symptoms over a very long follow-up period (up to 20 years). Of note, similar functional improvements persisted over time also in patients with severe left ventricular dilation and dysfunction at the time of diagnosis.

In the present study, a 1:2 propensity-matched comparison was performed among TIMIC patients and those receiving conventional therapy. In the latter group, cardiac function did not significantly improve, and patients had a higher incidence of death, cardiac transplantation, and the need for ICD implantation. These data are in agreement with a previous study reporting 10-year follow-up outcomes in patients with virus-negative chronic myocarditis or inflammatory cardiomyopathy treated with immunosuppression; this study showed a correlation between long-term functional improvement and normalization of the inflammatory process at histology.^9^

There is another important finding of our study that deserves further consideration. Specifically, the effectiveness of the TIMIC treatment was also demonstrated in patients who were initially allocated to the placebo arm of the trial and were subsequently switched to immunosuppressive therapy at study completion as a result of the documented superiority of immunosuppressive therapy. This observation is suggestive of a long-lasting persisting focal myocardial inflammation that does not resolve spontaneously or with supportive therapy alone.

The adoption of immunosuppression had important prognostic implications, since most treated patients did not experience new hospitalizations over time and progressively down-titrated the prescribed HF supportive treatment. This led to a significant cost reduction for the National Health System, especially since most patients were young with a long life expectancy. Mortality, need for heart transplantation, or ICD implantation occurred in a small number of patients who did not respond to immunosuppression (*Structured Graphical Abstract*).

### Recurrence of myocarditis

Among TIMIC patients, 6% experienced a recurrence of myocardial inflammation at long-term follow-up. Cardiac function worsening was paralleled by the evidence of a reactivation of the inflammatory process in the absence of myocardial viral genomes documented via cardiac MRI and histology. Four of these five patients with relapsing myocardial inflammation had an associated autoimmune manifestation, such as Hashimoto thyroiditis and autoimmune thrombocytopenia. These findings suggest that some individuals can be more susceptible to an inflammatory myocardial immune-mediated process that an unknown trigger can reactivate, similar to what happens in patients with systemic autoimmune diseases^21^ and that a close follow-up should be reserved for this cohort. Of note, all these patients showed cardiac functional recovery once immunosuppressive therapy was prescribed at the time of the index episode of myocarditis; similar beneficial effects occurred at the time of relapse once a new 6-month cycle of immunosuppression at the same dosage was started. Thus, immunosuppressive treatment can be safely repeated if a recurrence of virus-negative immune-mediated myocarditis occurs.

### Limitation of the study

Cardiac MRI was performed on a limited number of patients as this study was conducted at a time when this imaging technique was poorly available. Nevertheless, cardiac MRI was performed in all patients with recurrence of myocarditis before and after the application of the TIMIC protocol and showed the resolution of oedema and hyperaemia at follow-up, with persistence in the areas of fibrosis as suggested by late gadolinium enhancement.

## Conclusion

Virus-negative inflammatory cardiomyopathy may benefit from immunosuppressive therapy also after long-term follow-up. Recurrences of virus-negative myocardial inflammation appear to respond to a new TIMIC protocol application.

## Data Availability

Data are available on request.
